# Metformin Radiosensitizes p53-Deficient Colorectal Cancer Cells through Induction of G2/M Arrest and Inhibition of DNA Repair Proteins

**DOI:** 10.1371/journal.pone.0143596

**Published:** 2015-11-23

**Authors:** Youn Kyoung Jeong, Mi-Sook Kim, Ji Young Lee, Eun Ho Kim, Hunjoo Ha

**Affiliations:** 1 Graduate School of Pharmaceutical Sciences, College of Pharmacy, Ewha Womans University, Seoul, South Korea; 2 Research Center for Radiotherapy, Korea Institute of Radiological and Medical Sciences, Seoul, South Korea; 3 Department of Radiation Oncology, Korea Institute of Radiological and Medical Sciences, Seoul, South Korea; 4 Division of Heavy Ion Clinical Research, Korea Institute of Radiological and Medical Sciences, Seoul, South Korea; University Medical Center Hamburg-Eppendorf, GERMANY

## Abstract

The present study addressed whether the combination of metformin and ionizing radiation (IR) would show enhanced antitumor effects in radioresistant p53-deficient colorectal cancer cells, focusing on repair pathways for IR-induced DNA damage. Metformin caused a higher reduction in clonogenic survival as well as greater radiosensitization and inhibition of tumor growth of p53^-/-^ than of p53^+/+^ colorectal cancer cells and xenografts. Metformin combined with IR induced accumulation of tumor cells in the G2/M phase and delayed the repair of IR-induced DNA damage. In addition, this combination significantly decreased levels of p53-related homologous recombination (HR) repair compared with IR alone, especially in p53^-/-^ colorectal cancer cells and tumors. In conclusion, metformin enhanced radiosensitivity by inducing G2/M arrest and reducing the expression of DNA repair proteins even in radioresistant HCT116 p53^-/-^ colorectal cancer cells and tumors. Our study provides a scientific rationale for the clinical use of metformin as a radiosensitizer in patients with p53-deficient colorectal tumors, which are often resistant to radiotherapy.

## Introduction

Radiotherapy is widely used for the definitive and adjuvant treatment of numerous cancers [1]. However, resistance to radiotherapy remains an important concern [2]. Various factors including p53 mutation [3], overexpression of DNA repair proteins [4–6], and tumor microenvironment [7, 8] have been proposed to play roles in radioresistance. Among the radioresistant factors, p53 mutation is regarded as good candidate for radioresistance markers [9].

The tumor suppressor factor p53, which plays a central role in the cellular responses to DNA damage, promotes cell survival (cell-cycle arrest, DNA repair, and autophagy) at low levels of DNA damage while it induces cell death at high levels. Mutation of p53 occurs in more than 50% of human cancers, which significantly increases cellular resistance to γ radiation [10]. Additionally, p53 mutation correlates with high levels of DNA repair proteins including Rad51, which plays a key role in the DNA homologous recombination (HR) repair pathway. In addition, Rad51 is up-regulated in numerous cancers, especially high grade radioresistant tumors [11]. The HR repair pathway is modulated by p53-induced transcriptional repression of the *Rad51* gene and abrogation of Rad51 polymerization on DNA [12]. For this reason, radioresistance correlates with overexpression of Rad51 [13] while its downregulation conversely increases radiosensitivity [14]. Therefore, a promising approach to enhancing the efficacy of radiotherapy in patients with p53 mutant cancers is the discovery and use of DNA repair inhibitors as radiosensitizers.

Metformin, an oral biguanide anti-hyperglycemic agent, reportedly enhances responses to radiation by activating ataxia telangiectasia mutated (ATM)-adenosine monophosphate kinase (AMPK)-p53/p21^cip1^, which leads to apoptosis and inhibition of clonogenic survival [15, 16] in certain cancers. In addition, the combination of metformin and ionizing radiation (IR) enhanced the cytotoxic effects of IR in human hepatoma cell lines, which blocked the G2/M phase and decreased DNA repair by reducing adenosine triphosphate (ATP) production [17]. Interestingly, Buzzai *et al*. [18] reported that metformin selectively impaired cell growth in p53-deficient tumor cells by inhibiting autophagy, but activated autophagy in p53 wild-type tumor cells. Furthermore, several studies have shown that metformin increases radiosensitivity in p53-mutant or p53 wild-type cancer cells. Studies have also reported that metformin enhanced the radiosensitivity of p53-mutant [19] and p53 wild-type cancer cells. Furthermore, another study showed that metformin induced a moderate radioprotection [20]. However, there is no clear understanding of the differential effects of metformin combined with radiotherapy in p53-deficient and p53 wild-type cancer cells.

Therefore, this present study investigated whether metformin combined with IR would enhance the antitumor effects in radioresistant p53-deficient colorectal cancer cells. Furthermore, we focused on the possible involvement of the repair pathways for IR-induced DNA damage. We believe our data may contribute to providing a scientific rationale for the clinical use of metformin as a radiosensitizer in radioresistant cancers.

## Materials and Methods

### Materials

Metformin (1-(diaminomethylidene)-3, 3-dimethylguanidine), crystal violet, and propidium iodide were obtained from Sigma-Aldrich Chemical Corp., (St. Louis, MO, USA). Anti-Rad51, anti-Rad52, anti-excision repair cross-complementation group 1 (ERCC1), anti-breast cancer 2, early onset (BRCA2), and anti-phosphorylated-histone H3 (Ser10) antibodies were purchased from Abcam (Cambridge, MA, USA). Anti-meiotic recombination 11 (MRE11), anti-Rad50, anti-p95/ Nijmegen breakage syndrome protein 1 (NBS1), anti-BRCA1, anti-cyclinB1, anti-phospho- cell division cycle protein 2 homolog (cdc2, Tyr15), and anti-phospho- checkpoint kinase 2 (Chk2, Thr68) were obtained from Cell Signaling Technology (Beverly, MA, USA). Anti-p53, anti-Chk2, anti-*β*-actin, and horseradish peroxidase (HRP)-conjugated goat anti-rabbit and anti-mouse IgG antibodies were purchased from Santa Cruz Biotechnology (Santa Cruz, CA, USA). Anti- H2A histone family, member X (H2AX, Ser139) was provided by Millipore (Billerica, MA, USA). Alexa Fluor 488 goat anti-mouse IgG (H+L) and Alexa Fluor 594 goat anti-rabbit IgG (H+L) secondary antibodies were purchased from Invitrogen (Carlsbad, CA, USA). Fluorescence mounting medium was obtained from Dako (Glostrup, Denmark). Roswell Park Memorial Institute (RPMI) 1640 medium, fetal bovine serum (FBS), and antibiotics (penicillin and streptomycin) were obtained from Lonza (Walkersville, MD, USA).

### Cell lines and cell culture

HCT116 p53^+/+^ human colorectal cancer cells were obtained from the Korean Cell Line Bank (Seoul, South Korea) and HCT116 p53^-/-^ human colorectal cancer cells were kindly provided by Dr. B. Vogelstein of Johns Hopkins University. HCT116 p53^+/+^ and p53^-/-^ cells were cultured in RPMI 1640 supplemented with 10% FBS, 100 units/mL penicillin, and 100 μg/mL streptomycin and incubated in an atmosphere of 5% CO_2_ at 37°C.

### Irradiation

For *in vitro* experiments, the cells were irradiated with a ^137^Cs γ-ray source (Atomic Energy of Canada, Ltd., Chalk River, Ontario, Canada) at a dose rate of 2.67 Gy/min. For *in vivo* experiments, mice were irradiated using a ^60^Co γ-ray source (Theratron 780, Atomic Energy of Canada, Chalk River, Ontario, Canada) with a 0.5 cm diameter bolus of tissue equivalent material to allow for dose buildup. A lead barrier was used to shield normal tissues where possible.

### Water-soluble tetrazolium (WST-1) assay

For the cytotoxicity assay, cells were seeded in 96-well culture plastic plates at a density of 1 × 10^3^ cells per well. Metformin at varying concentrations (0–10 mM) was added to each well and the cells were incubated for 48 h followed by application of the water-soluble tetrazolium (WST)-1 cytotoxicity assay reagent (Roche Diagnostics, Laval, Quebec, Canada) according to the manufacturer’s recommendations. Cell viability was assessed by determining the A450 nm of the cell culture media after the addition of WST-1 for 2 h. The results were reported as a percentage of the optical density of the untreated control cells, which was designated as 100% cell viability. Percentage of cytotoxicity was calculated as follows: (1-Aexp /Acon) × 100; where Aexp and Acontrol are the absorbance values of the experimental drug-treated and control un-treated cells, respectively.

### Clonogenic assay

HCT116 p53^+/+^ and p53^-/-^ cells were treated with metformin at 1–10 mM for 48 h or 2.5 mM for 24 h followed by IR, and then further incubated for 24 h. The clonogenic assay was then conducted as previously described [17].

### Tumor xenografts in athymic mice

Athymic Balb/c nude mice (4-week-old males) were obtained from Nara Biotech Co. (Seoul, Korea) and maintained in a laminar airflow cabinet under specific pathogen-free conditions. HCT116 p53^+/+^ and p53^-/-^ xenograft mouse models were established by subcutaneous inoculation of 3 × 10^6^ HCT116 p53^+/+^ or p53^-/-^ cells into the right hind leg. After tumor implantation, we monitored the condition of the animals once a day and prepared analgesics to minimize suffering of the animals. When the tumor attained a volume of about 100 mm^3^, the mice were randomly divided into four groups (n = 5) including (a) control, (b) metformin, (c) IR, and (d) combination of metformin and IR. The metformin-treated groups (b and d) were injected (intraperitoneally) once a day with 250 mg/kg.

When the tumor volume of the control group attained 200 mm^3^, the IR-treated groups (c and d) were treated with a single 5 Gy fraction of local-regional irradiation using a ^60^Co irradiator. The tumor volume (V) was calculated using the standard formula: V (mm^3^) = π/6 × (smaller diameter)^2^ × (larger diameter). Mice were euthanized by carbon dioxide (CO_2_) inhalation when the average tumor volume of the control group was 1,000 mm^3^.

### Cell cycle analysis

After metformin (2.5 mM) exposure for 24 h, cells were irradiated, incubated for 24 h or 48 h, harvested, stained with propidium iodide (1 mg/mL) according to the manufacturer’s protocol, and then analyzed using a FACScan flow cytometer (Becton Dickinson, Franklin Lakes, NJ, USA). A minimum of 10,000 cells were counted for each sample and data analysis was performed using the CellQuest software.

### Immunofluorescence

Immunofluorescence staining was performed to determine the nuclear distribution of γ-H2AX and Rad51 in HCT116 p53^+/+^ and p53^-/-^ cells using image analysis in the three (green/red/blue) fluorescence channels. Cells were grown on chambered slides 1 day prior to irradiation or metformin treatments. After metformin (2.5 mM) exposure for 24 h, cells were irradiated and incubated for 1 h or 24 h. All treatments were performed while cells remained attached to the slides. Cells were fixed (4% paraformaldehyde in phosphate-buffered saline, PBS, 10 min), permeabilized (0.5% Triton X-100 in PBS, 10 min), and incubated with blocking buffer (4% FBS in PBS, 1h) and then incubated overnight at 4°C or for more than 4h at room temperature with primary antibodies (1:100 each, anti-γ-H2AX and anti-Rad51), Then, the cells were washed with PBS, incubated for 1 h at room temperature in the dark, with appropriate fluorescein isothiocyanate (FITC)-labeled secondary antibodies (1:500 each) including Alexa Fluor 488 goat anti-mouse IgG (H+L) for γ-H2AX (green) and Alexa Fluor 594 goat anti-rabbit IgG (H+L) for Rad51(red). The cells were then washed with PBS, stained with DAPI (blue), and mounted onto slides using fluorescence mounting medium. The slides were finally examined using a Carl Zeiss LSM510 laser scanning microscope and images were captured with a charge coupled device camera. For quantitative analysis, γ-H2AX or Rad51 foci-positive cells were counted in at least 100 cells from randomly captured images.

### Immunoblotting

The cells or tumor tissues were lysed with a radioimmunoprecipitation assay (RIPA) buffer and the proteins were separated using sodium dodecyl sulfate (SDS) polyacrylamide gel electrophoresis (PAGE) and transferred to nitrocellulose membranes. The membranes blots were blocked with 5% (v/v) skim milk in PBS with 0.1% Tween 20, incubated with the indicated antibodies (1:1,000) and secondary antibodies (1:1,000), and then subsequently developed using enhanced chemiluminescence western blotting substrate (Pierce, Rockford, IL, USA) using the ImageQuant LAS-4000 mini (GE, Fairfield, CT, USA). The signal intensity of the bands was measured with the ImageJ program (National Institutes of Health, NIH, Bethesda, MD, USA).

### Immunohistochemistry

For immunohistochemical analysis, 4-μm tumor tissue sections were de-paraffinized with xylene and sequentially rehydrated in a series of graded concentrations of ethanol. To reduce nonspecific background staining due to endogenous peroxidase, the slides were blocked in hydrogen peroxide for 10 min, and washed four times in buffer. The ERCC1 (1:50) and Rad51 (1:200) primary antibodies were applied to the tissue slides, which were incubated according to the manufacturers’ protocols, and then washed four times in buffer. The slides were incubated with primary antibody enhancer for 20 minutes at room temperature, and washed four times in buffer. Then, the HRP polymer was applied to the slides and the color was developed using the Zymed diaminobenzidine (DAB) system (Zymed Laboratories, Inc., South San Francisco, CA, USA) followed by counterstaining with hematoxylin. The histological scores for Rad51 and ERCC1 expression were determined using two independent methods: percentage positive staining cells and expression intensity.

### Statistical analysis

All data were expressed as the mean ± standard error of the mean (S.E.M.). The statistical analysis was performed using a parametric repeated measure one-way analysis of variance (ANOVA) followed by Tukey honest significant difference (HSD) test using the statistical package for the social sciences (SPSS) software (version 18.0, Chicago, IL, USA). Statistical significance was set at a level of *p* < 0.05.

### Ethics statement

All animal study protocols and studies were approved by the Institutional Animal Care and Use Committee (IACUC) of the Korean Institute of Radiological and Medical Sciences (KIRAMS 2013–57).

## Results

### Metformin differentially decreased p53-deficient and wild type clonogenic cell survival

**Fig 1:**
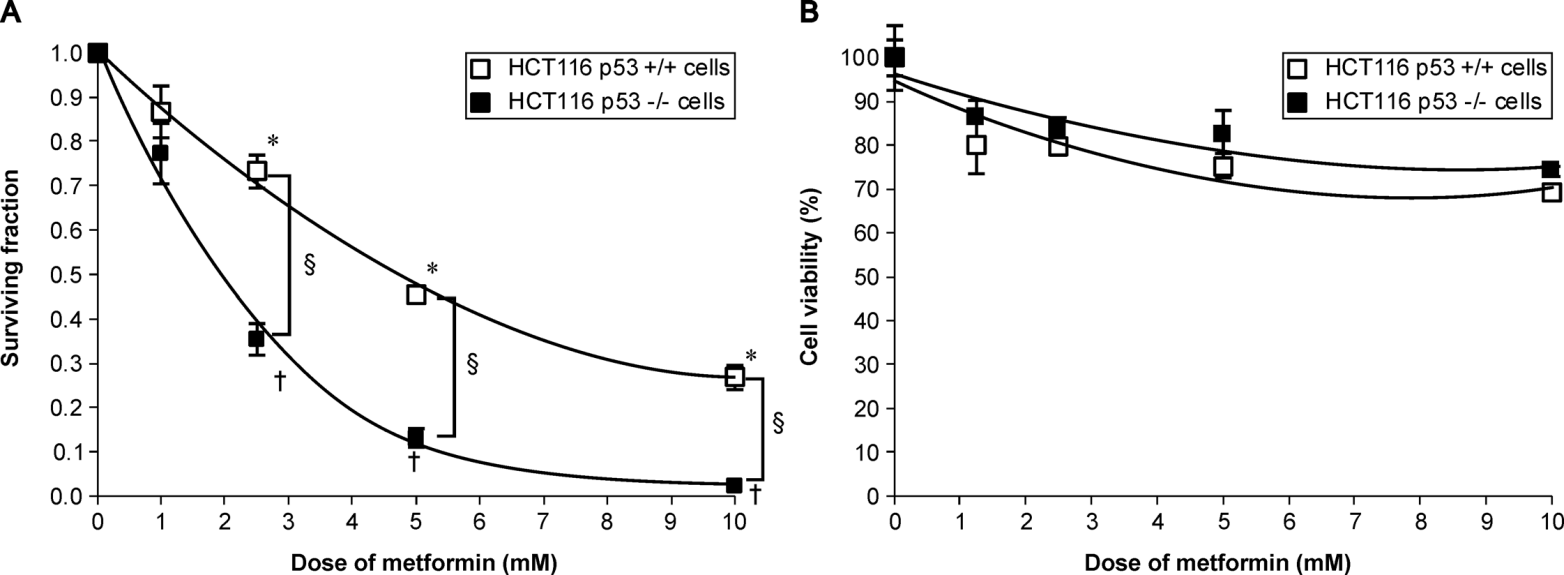
Metformin decreased clonogenic survival in p53-deficient compared with p53 wild-type cells. HCT116 p53^+/+^ and p53^-/-^ cells were treated with 1–10 mM metformin for 48 h, cultured and used in (A) clonogenic and (B) WST-1 (cell viability) assays; *p < 0.001 for HCT116 p53^+/+^ cells, ^†^ *p* < 0.001 for HCT116 p53^-/-^ cells. ^§^ *p* < 0.001, between HCT116 p53^+/+^ and p53^-/-^ cells, compared to control. WST, water-soluble tetrazolium.

To evaluate metformin-p53-dependent clonogenicity and cytotoxicity in human colorectal cancer cells, we exposed HCT116 p53^+/+^ and p53^-/-^ cells to varying concentrations for 48 h. Metformin at 1, 2.5, 5, and 10 mM showed 86.7±5.9, 73.2±3.8, 45.5±1.1, and 26.8±2.7% clonogenic survival rates for HCT116 p53^+/+^ cells, and 77.2±6.9, 35.5±3.6, 13.1±2.1, and 2.3±0.9% for HCT116 p53^-/-^ cells, respectively (Fig 1A). Clonogenic survival of HCT116 p53^+/+^ and p53^-/-^ cells differed significantly at metformin concentrations 2.5–10 mM (*p* < 0.001), and HCT116 p53^-/-^ cells were more sensitive to metformin-induced clonogenic cell death than HCT116 p53^+/+^ cells were. However, metformin had no significant or differential cytotoxicity against both cell lines (Fig 1B).

### Metformin induced higher radiosensitization in p53-deficient than p53 wild-type cells

**Fig 2:**
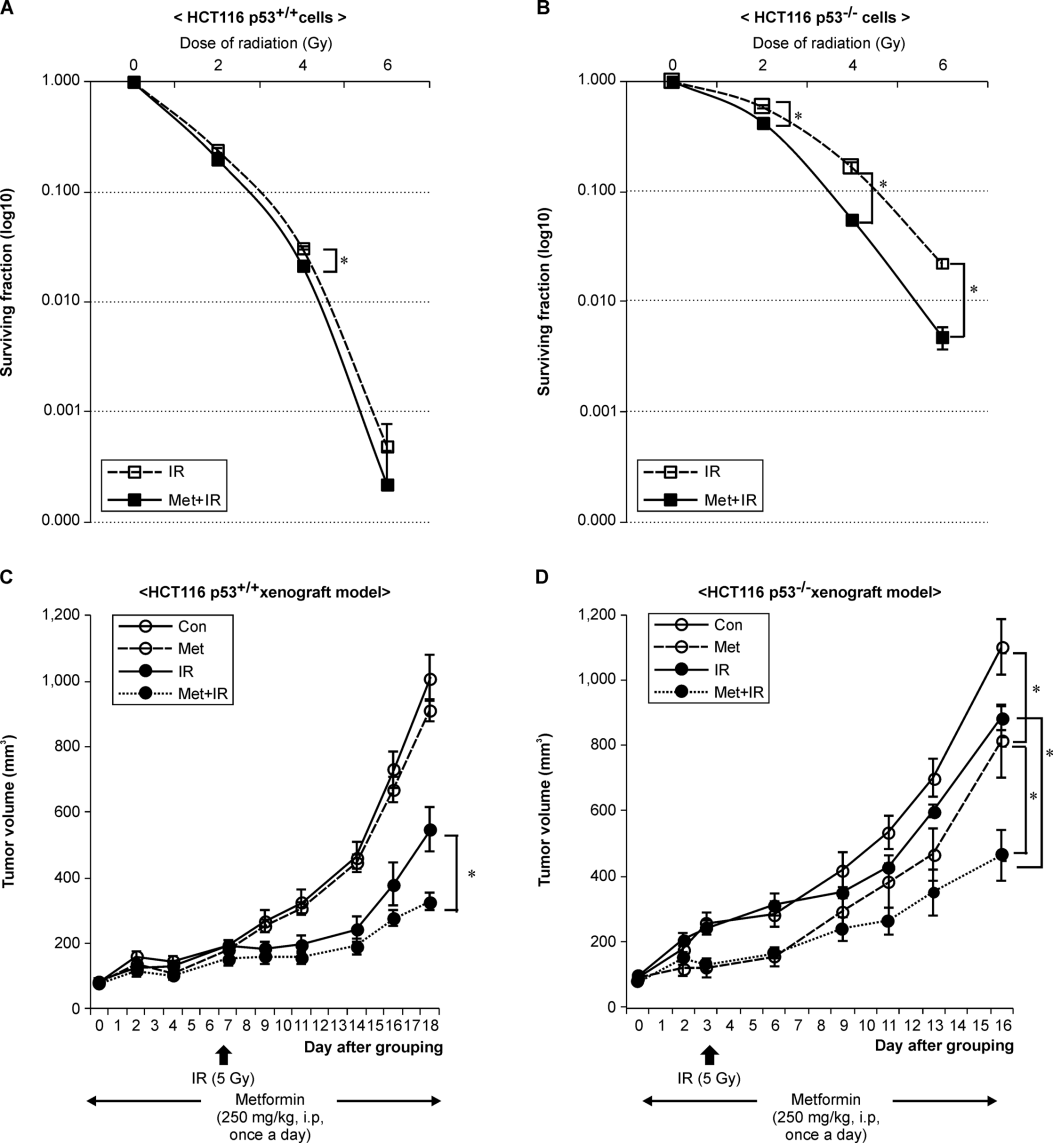
Metformin significantly inhibited tumor growth and enhanced radiosensitivity in p53-deficient cells and xenografts. Radiosensitivity of (A) HCT116 p53^+/+^ and (B) p53^-/-^ cells with and without exposure to metformin (2.5 mM) after varying doses of ^60^Co γ-ray radiation was measured using a clonogenic assay. HCT116 p53^+/+^ and p53^-/-^ xenografted mice were randomly divided into four groups including control (Con), metformin (Met), ionizing radiation (IR), and combination of metformin and IR (Met+IR). Graphs indicate the tumor volume in (C) HCT116 p53^+/+^ and (D) p53^-/-^ xenograft model; *p < 0.05.

To assess the radiosensitizing effects of metformin, HCT116 p53^+/+^ and p53^-/-^ cancer cells were treated for 24 h with 2.5 mM followed by IR, and then removed 24 h after IR. The surviving fractions following exposure to a 2 Gy radiation dose (SF2) were determined to be 0.24 and 0.20 for HCT116 p53^+/+^ cells (Fig 2A) and 0.58 and 0.43 for HCT116 p53^-/-^ cells (Fig 2B), following treatment with radiation alone and metformin plus radiation, respectively. The combination of metformin and IR markedly reduced clonogenic survival compared with IR alone in HCT116 p53^-/-^ (*p* < 0.01), but not p53^+/+^ cells, suggesting that radiosensitization was higher in HCT116 p53^-/-^ than in p53^+/+^ cells.

### Metformin inhibited tumor growth in p53-deficient xenografts

To investigate the effects of metformin on p53-dependent and independent tumor growth, we used paired isogenic human colorectal HCT116 p53^+/+^ and p53^-/-^ cancer cells. When the mean of the tumor volume in the control group attained about 1,000 mm^3^, the xenografts of the tumor volume in mice treated with metformin, IR, and a combination of both were 4.5±0.2, 48.3±6.1, and 59.0±5.0% smaller, respectively, than that of the controls at about 1,000 mm^3^ for HCT116 p53^+/+^ tumors (Fig 2C) and 26.5±1.1, 22.8±2.9, and 44.3±5.5%, respectively for HCT116 p53^-/-^ tumors (Fig 2D).

The growth of the control HCT116 p53^-/-^ xenografts was significantly faster than that of the HCT116 p53^+/+^ xenografts was (*p* < 0.05). The combined metformin and IR treatment significantly inhibited the tumor growth of both xenograft models more than IR treatment alone did (*p* < 0.05). Furthermore, metformin enhanced antitumor effects in p53^-/-^ (*p* < 0.05) but not p53^+/+^ xenografts while IR alone markedly suppressed tumor growth in p53^+/+^ xenografts (*p* < 0.05).

### Metformin prolonged IR-induced G2/M arrest in p53-deficient colorectal cells compared with p53 wild- type cells

**Fig 3:**
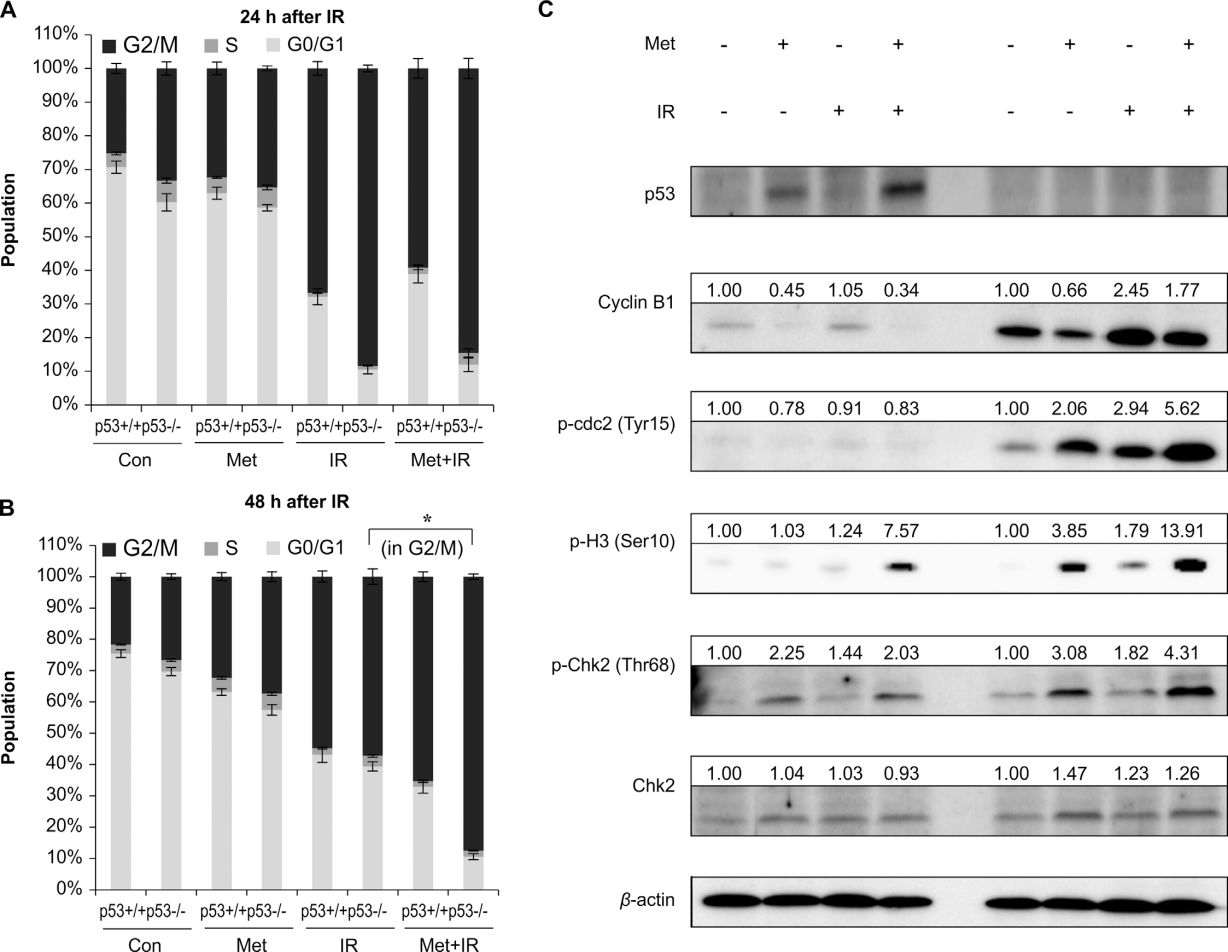
Metformin prevented cell cycle progression by significantly prolonging IR-induced G2/M arrest in HCT116 p53^-/-^ cells. HCT116 p53^+/+^ and p53^-/-^ cells were pretreated with 2.5 mM metformin for 24 h, and then irradiated with 6 Gy. Cell cycle measured by flow cytometry (A) 24 and (B) 48 h after IR. (C) Expression of G2/M checkpoint regulators including cyclin B1, phosphorylated cdc2 (Tyr15), phosphorylated histone H3 (Ser10), and phosphorylated Chk2 (Thr68) were measured using immunoblotting in HCT116 p53^+/+^ and p53^-/-^ cells 48 h after irradiation. Densitometric quantification was normalized to *β*-actin. Values are mean ± SEM. of three experiments, *p < 0.001. IR, ionizing radiation

To determine whether metformin affected IR-induced cell cycle accumulation in the G2/M phase and the proportion of G2/M phase cells associated in the presence or absence of p53, we analyzed the cell cycle phase distribution of HCT116 p53^+/+^ and p53^-/-^ cells. Metformin combined with IR significantly increased the proportion of G2/M phase cells compared with IR alone in HCT116 p53^-/-^ (*p* < 0.001), but not p53^+/+^ cells (Fig 3A and 3B).

To confirm the accumulation of G2/M phase, the expression levels of G2/M checkpoint regulators such as cyclin B1, phosphorylated cdc2 (Tyr15), phosphorylated histone H3 (Ser10), and phosphorylated Chk2 (Thr68) were examined using immunoblotting. The combination of metformin and IR significantly increased the expression of phosphorylated cdc2 (Tyr15) compared with IR alone in HCT116 p53^-/-^ but not p53^+/+^cells (*p* < 0.05). Furthermore, metformin plus IR markedly increased the expression of phosphorylated histone H3 (Ser10) and phosphorylated Chk2 (Thr68) compared with IR alone in HCT116 p53^+/+^ and p53^-/-^ cells. In addition, the expression of phosphorylated histone H3 (Ser10) and phosphorylated Chk2 (Thr68) in the metformin plus IR group was significantly increased in HCT116 p53^-/-^ compared with p53^+/+^ cells (*p* < 0.001, Fig 3C). In summary, we demonstrated that metformin prevented cell cycle progression by significantly prolonging IR-induced G2/M arrest in HCT116 p53^-/-^ cells.

### Metformin delayed repair of IR-induced DNA damage in p53-deficient cells compared to that in p53 wild-type cells

**Fig 4:**
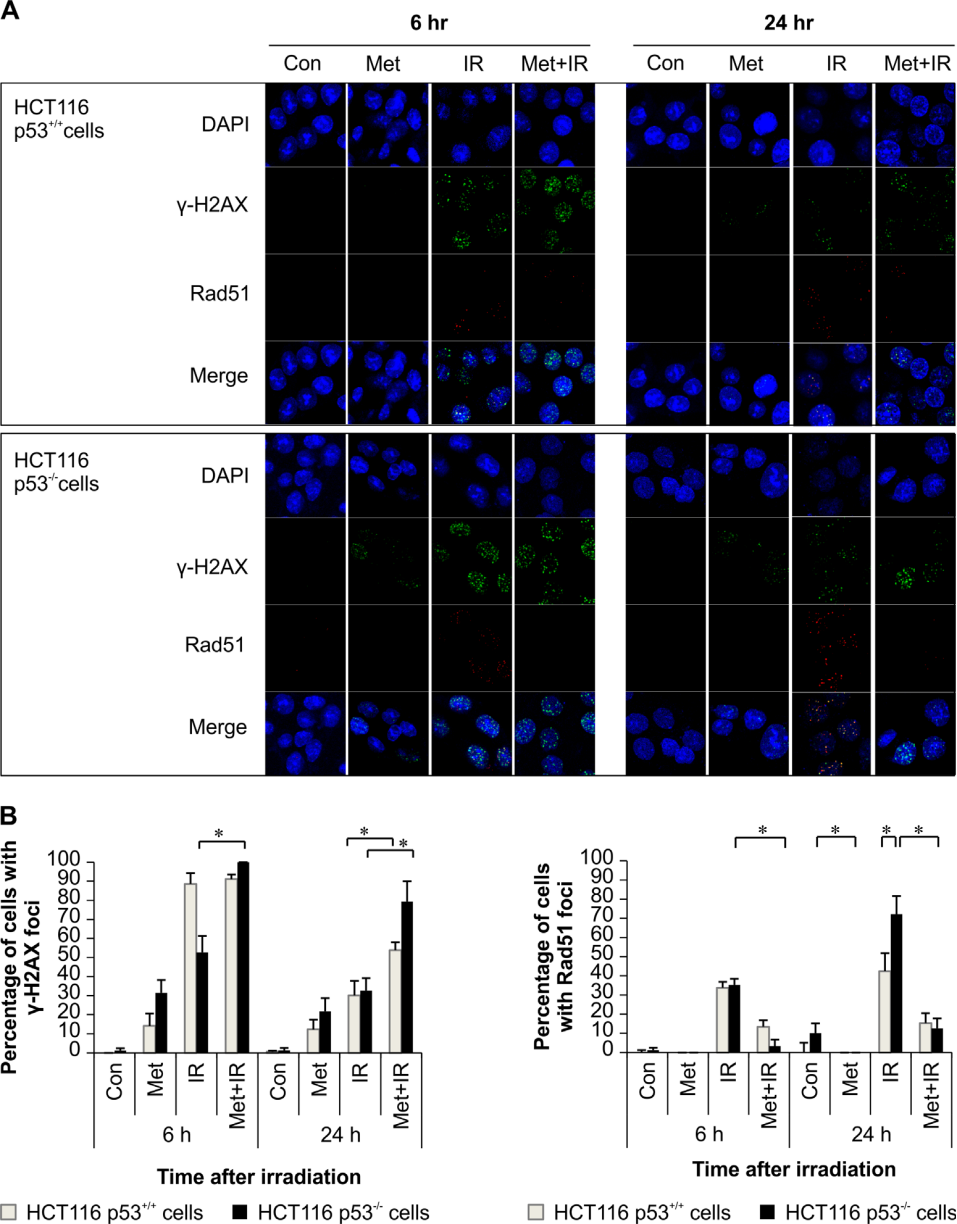
Metformin delayed repair of IR-induced DNA damage in p53-deficient cells compared with p53 wild-type cells. HCT116 p53^+/+^ and p53^-/-^ cells were pretreated with 2.5 mM metformin for 24 h, and then irradiated with 6 Gy. (A) Immunofluorescence staining at 6 and 24 h after IR, showed γ-H2AX, a marker for DNA damage; Rad51, a marker of DNA repair; and nuclear DNA stained with DAPI using image analysis in three (green/red/blue) fluorescence channels. (B) For quantitative analysis, γ-H2AX or Rad51 foci-positive cells were counted in at least 100 cells from randomly captured images. Values are mean ± S.E.M, *p < 0.001. IR, ionizing radiation; γ-H2AX, γ-H2A histone family, member X.

We analyze the effect of metformin on the kinetics of DNA damage and repair by assessing the immunofluorescent staining for γ-H2AX, a marker for DNA damage; Rad51, a marker of DNA repair; and DAPI-stained nuclear DNA using image analysis in the three (green/red/blue) fluorescence channels. Metformin combined with IR and IR alone exhibited γ-H2AX foci at 6 h after IR while metformin combined with IR retained the γ-H2AX foci for up to 24 h after IR. In addition, this effect was greater in HCT116 p53^-/-^ than it was in p53^*+/+*^ cells (*p* < 0.001). However, 6 and 24 h after IR, metformin combined with IR showed a significant decrease in the Rad51 foci compared with IR alone, which showed an increase in the Rad51 foci. (Fig 4A and 4B). HCT116 p53^-/-^ cells were more sensitive to metformin than HCT116 p53^+/+^ cells (*p* < 0.001). These results showed that the combination of metformin and IR delayed the repair of IR-induced DNA damage more in HCT116 p53^-/-^ than in p53^*+/+*^ cells.

### Metformin enhanced radiosensitivity by reducing DNA repair proteins *in vitro* and *in vivo*

**Fig 5:**
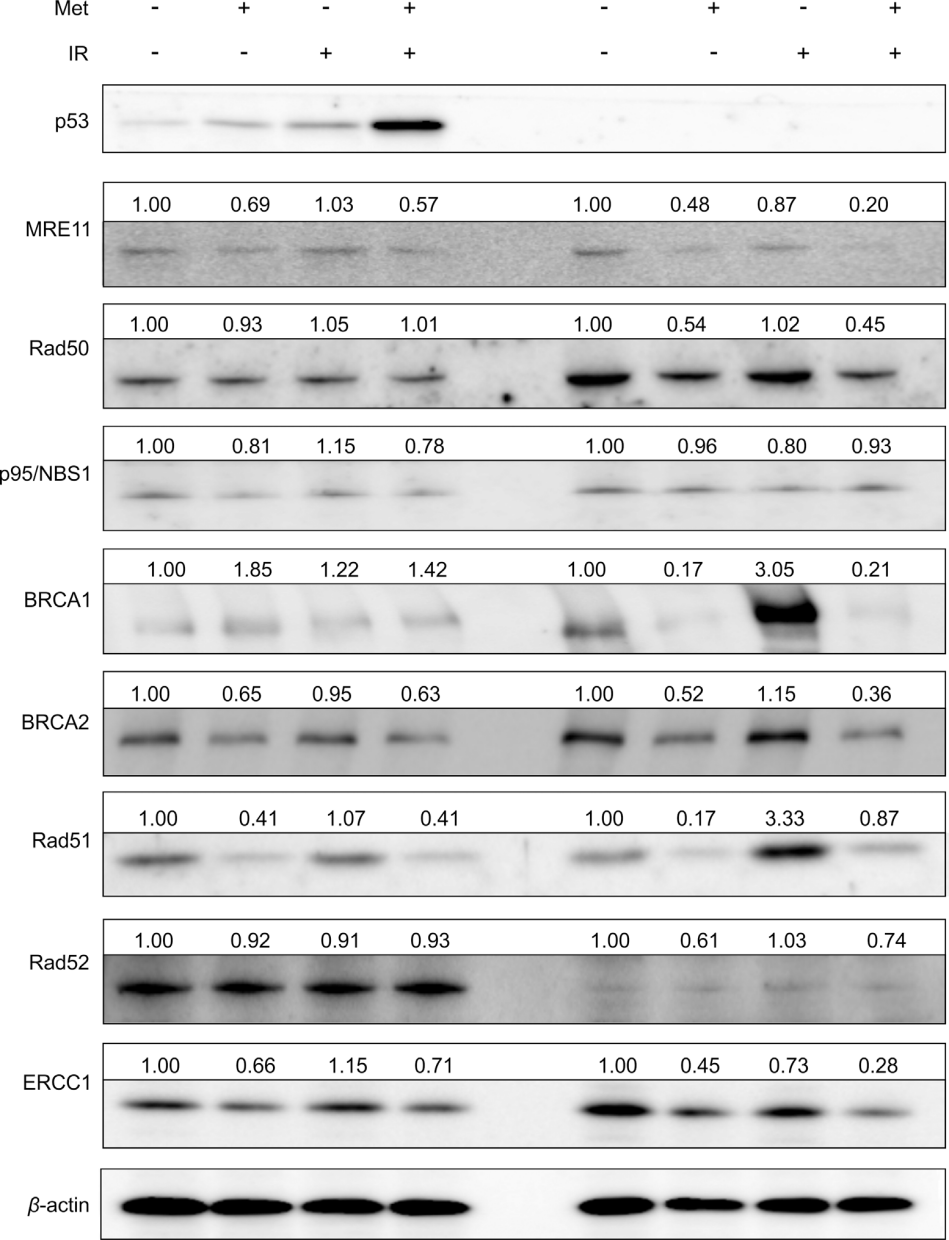
Metformin enhanced radiosensitivity by reducing p53-related HR repair proteins *in vitro*, especially in p53-deficient cells. HCT116 p53^+/+^ and p53^-/-^ cells were pretreated with 2.5 mM metformin for 24 h, and then irradiated with 6 Gy. Cells from both groups were immunoblotted with p53-related HR repair proteins such as MRE11-Rad50-p95/NBS1 complex, BRCA1, BRCA2, Rad51, Rad52, and ERCC1 24 h after ionizing radiation (IR). Densitometric quantification was normalized to *β*-actin. HR, homologous recombination; MRE11, meiotic recombination 11; NBSI, nijmegen breakage syndrome protein 1; BRCA, breast cancer early onset; ERCC 1, excision repair cross-complementation group 1.

To investigate whether metformin affected DNA repair pathways *in vitro*, we examined the expression of p53-related HR repair proteins including the MRE11-Rad50-p95/NBS1 complex, BRCA1, BRCA2, Rad51, Rad52, and ERCC1 in HCT116 p53^+/+^ and p53^-/-^ cells using immunoblotting. As shown in Fig 5, metformin combined with IR markedly reduced MRE11, BRCA2, Rad51, and ERCC1 protein levels compared with IR alone in HCT116 p53^+/+^ and particularly p53^-/-^ cells (*p* < 0.01). Moreover, metformin combined with IR significantly decreased the expression of Rad50 and BRCA1 compared with IR alone in HCT116 p53^-/-^ but not p53^+/+^cells (*p* < 0.01). Interestingly, in HCT116 p53^-/-^ cells, IR alone significantly increased BRCA1 and Rad51 protein levels compared with the control (*p* < 0.01) while metformin combined with IR markedly decreased the expression of BRCA1 and Rad51 compared with IR alone (*p* < 0.01).

**Fig 6:**
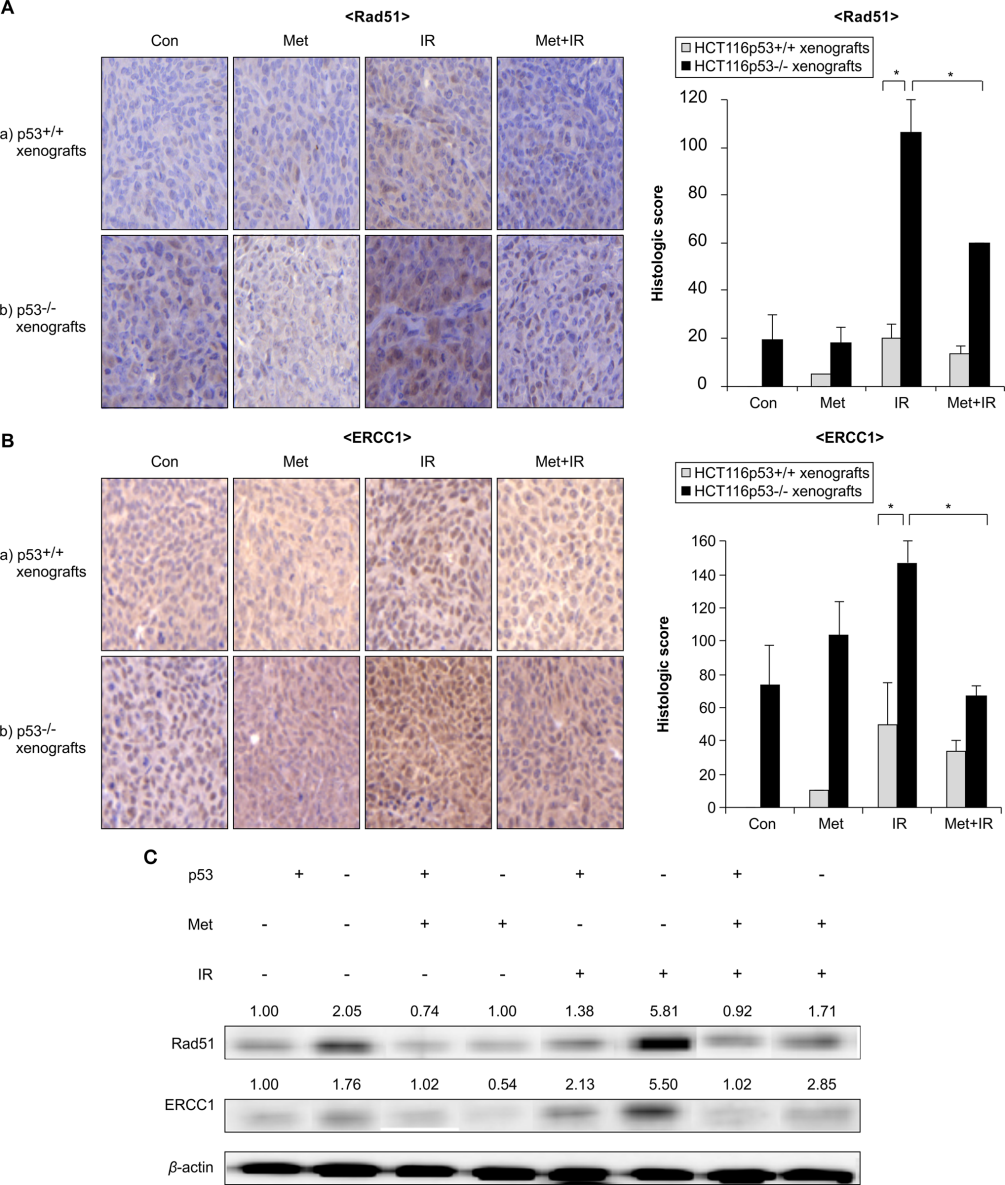
Metformin enhanced radiosensitivity by reducing Rad51 and ERCC1 proteins *in vivo*, especially in p53-deficient xenografts. *In vivo*, confirmation of (A) Rad51 and (B) ERCC1 expression in HCT116 p53^+/+^ and p53^-/-^ tumor tissues analyzed by immunohistochemistry and graphs indicate histological score for each group. (C) Tumor tissues were lysed and immunoblotted with Rad51 and ERCC1. Densitometric quantification was normalized to *β*-actin. Data are mean ± S.E.M, *p < 0.05.

Next, we evaluated whether metformin affected DNA repair pathways *in vivo* by immunohistochemically examining the expression of Rad51 and ERCC1 in tumor samples. Rad51 and ERCC1 protein levels of the HCT116 p53^-/-^ control group tumors were high but were below the limits of detection for the p53^+/+^ control group tumors. The expression of IR-induced Rad51 and ERCC1 was markedly increased in HCT116 p53^-/-^ tumors compared with p53^+/+^ tumors while metformin combined with IR significantly decreased the protein levels of Rad51 and ERCC1 in HCT116 p53^-/-^ compared with p53^+/+^ tumors (*p* < 0.05, Fig 6A and 6B). Additionally, we confirmed the expression of Rad51 and ERCC1 by immunoblotting tumor samples from the *in vivo* study. The IR-induced Rad51 and ERCC1 expression was more markedly increased in HCT116 p53^-/-^ than it was in p53^+/+^ tumors (*p* < 0.05, Fig 6C). In addition, metformin combined with IR significantly decreased the expression of Rad51 in HCT116 p53^-/-^ tumors (*p* < 0.05) to levels comparable to those in p53^+/+^ tumors (Fig 6C). In summary, we demonstrated that metformin enhanced tumor radiosensitivity by reducing DNA repair protein levels, especially in HCT116 p53^-/-^ cells and tumors.

## Discussion

Mutation of the tumor suppressor p53 factor plays a major role in the radioresistance of cancer cells [9]. More than 50% of all cancers have a missing or damaged p53 gene and, therefore, have the propensity to become highly radioresistant. In addition, p53 mutation correlated with high levels of DNA repair proteins [10]. Among the DNA repair proteins, p53 associated with HR by transcriptional repression of the *Rad51* gene and abrogation Rad51 polymerization on DNA [12]. Recently, the cytotoxic [21–23] and radiosensitizing [16, 17, 24, 25] effects of metformin were reported in various cancer cells without consideration of p53 status. However, Buzzai *et al*. [18] showed that metformin selectively impaired p53^-/-^ cells in the absence of glucose or in a solid tumor microenvironment. In this study, we investigated the ability of metformin to enhance IR-induced antitumor effects in radioresistant p53-deficient colorectal cancer cells, focusing on repair pathways for IR-induced DNA damage.

First, we confirmed that HCT116 p53^-/-^ xenografts not only grew faster but also were radioresistant and more sensitive to metformin than p53^+/+^ xenografts were. Metformin inhibited tumor growth and surmounted radioresistance in HCT116 p53^-/-^ xenografts. While metformin induced concentration-dependent cytotoxicity in both HCT116 p53^+/+^ and p53^-/-^ cells, this effect was greater in p53^-/-^ than it was in p53^+/+^ cells. Our previous study [24] showed that metformin induced apoptosis in hepatocellular carcinoma but not in normal hepatocytes.

Next, we investigated the radiosensitization mechanisms of metformin in HCT116 p53^+/+^ and p53^-/-^ cells and xenografts. Metformin combined with IR was recently reported to induce cytotoxicity of hepatoma cells more than IR alone did, by inhibiting DNA repair, which was mediated by the accumulation of cells in the cell cycle G2/M phase and disappearance of γ-H2AX expression [17]. The present results showed that metformin combined with IR prevented cell cycle progression by significantly prolonging IR-induced G2/M arrest, and induced greater DNA damage than IR alone did. In particular, the HCT116 p53^-/-^ cells were more sensitive to metformin than HCT116 p53^+/+^ cells were, suggesting that metformin delayed the repair of IR-induced DNA damage in HCT116 p53^-/-^ cells. Accordingly, these data suggest that the metformin-induced radiosensitization may be associated with DNA damage and repair pathways.

The tumor suppressor p53 protein regulates the expression of DNA repair proteins and numerous studies have reported that DNA repair proteins such as Rad51 [26, 27] and ERCC1 [28] were highly active in cancer cells that lack functional p53 but less active in normal cells and cancer cell lines with intact p53 function. In our *in vivo* studies, the expression of Rad51 and ERCC1 at basal levels and after IR were markedly higher in p53^-/-^ than they were in p53^+/+^ tumors (Fig 6), which is in agreement with previous reports [26–28]. Metformin combined with IR significantly decreased the protein levels of Rad51 and ERCC1 in p53^-/-^ tumors to levels that were comparable to those of p53^+/+^ tumors. Furthermore, our *in vitro* studies demonstrated that the combination of metformin and IR significantly reduced the expression of p53-related HR repair proteins such as MRE11, Rad50, BRCA1, BRCA2, Rad51, and ERCC1 compared with IR alone in HCT116 p53^+/+^ and particularly p53^-/-^ cells. Furthermore, IR alone significantly increased BRCA1 and Rad51 protein levels compared with control in HCT116 p53^-/-^ cells while metformin combined with IR markedly decreased the expression of BRCA1 and Rad51 compared with IR alone in HCT116 p53^-/-^ cells. Therefore, these results suggest that metformin enhances radiosensitivity in p53^-/-^ cells and tumors, which highly expressed BRCA1, Rad51, or ERCC1 protein in control and following IR treatment by reducing the expression of DNA repair proteins. The exact mechanism how metformin more reduces radiation induced-DNA repair proteins in p53 null than wild-type colorectal cancer cells remains further study. In this context, metformin inhibits mammalian target of rapamycin (mTOR) signaling in several cancer cells [29, 30]. Chang *et al*. [31] reported that mTOR inhibitors enhance radiosensitivity through inducing apoptosis, reducing autophagy, and suppressing DNA repair proteins in radioresistant prostate cancer cells. Buzzai *et al*. [18] also reported that metformin selectively impaired cell growth in p53 null colorectal cancer cells by inhibiting autophagy but activated autophagy in p53 wild-type colorectal cancer cells. It is, therefore, speculated that metformin may reduce DNA repair proteins possibly by inhibiting mTOR in p53 null colorectal cancer cells and enhance radiosensitivity.

In conclusion, metformin induced a concentration-dependent clonogenic cell death, inhibited tumor growth, and enhanced radiosensitivity by inducing G2/M arrest and inhibiting p53-related HR DNA repair proteins. In addition, these effects were greater in cells and xenografts of the HCT116 p53^-/-^ than p53^+/+^ cell lines. Finally, our results provide a scientific rationale for the clinical application of metformin as a radiosensitizer in patients with p53-deficient tumors, which are often resistant to chemotherapy or radiotherapy.
